# Promoting FAIR Data Through Community-driven Agile Design: the Open Data Commons for Spinal Cord Injury (odc-sci.org)

**DOI:** 10.1007/s12021-021-09533-8

**Published:** 2021-08-04

**Authors:** Abel Torres-Espín, Carlos A. Almeida, Austin Chou, J. Russell Huie, Michael Chiu, Romana Vavrek, Jeff Sacramento, Michael B. Orr, John C. Gensel, Jeffery S. Grethe, Maryann E. Martone, Karim Fouad, Adam R. Ferguson, the STREET-FAIR Workshop Participants, Warren Alilain, Mark Bacon, Nicholas Batty, Michael Beattie, Jacqueline Bresnahan, Emily Burnside, Sarah Busch, Randall Carpenter, Isaac Francos Quijorna, Xiaohui Guo, Agnes Haggerty, Sarah Haroon, Jack Harris, Lyn Jakeman, Linda Jones, Naomi Kleitman, Timothy Kopper, Michael Lane, Francisco Magana, David Magnuson, Ines Maldonado, Verena May, Katelyn McFarlane, Kazuhito Morioka, Martin Oudega, Philip Leo Pascual, Jean-Baptiste Poline, Ephron Rosenzweig, Emma Schmidt, Wolfram Tetzlaff, Lana Zholudeva

**Affiliations:** 1 Weill Institute for Neurosciences, Brain and Spinal Injury Center, Department of Neurological Surgery, University of California San Francisco, San Francisco, CA USA; 2 Department of Neuroscience, University of California, San Diego, San Diego, CA USA; 3 Faculty of Rehabilitation Medicine and the Neuroscience and Mental Health Institute, University of Alberta, Edmonton, AB Canada; 4 Spinal Cord and Brain Injury Research Center, Department of Physiology, University of Kentucky College of Medicine, Lexington, KY USA; 5 San Francisco Veterans Affairs Health Care System, San Francisco, CA USA

**Keywords:** Data sharing, FAIR, spinal cord injury, neurotrauma, data reuse, community repository

## Abstract

The past decade has seen accelerating movement from data protectionism in publishing toward open data sharing to improve reproducibility and translation of biomedical research. Developing data sharing infrastructures to meet these new demands remains a challenge. One model for data sharing involves simply attaching data, irrespective of its type, to publisher websites or general use repositories. However, some argue this creates a ‘data dump’ that does not promote the goals of making data Findable, Accessible, Interoperable and Reusable (FAIR). Specialized data sharing communities offer an alternative model where data are curated by domain experts to make it both open and FAIR. We report on our experiences developing one such data-sharing ecosystem focusing on ‘long-tail’ preclinical data, the Open Data Commons for Spinal Cord Injury (odc-sci.org). ODC-SCI was developed with community-based agile design requirements directly pulled from a series of workshops with multiple stakeholders (researchers, consumers, non-profit funders, governmental agencies, journals, and industry members). ODC-SCI focuses on heterogeneous tabular data collected by preclinical researchers including bio-behaviour, histopathology findings and molecular endpoints. This has led to an example of a specialized neurocommons that is well-embraced by the community it aims to serve. In the present paper, we provide a review of the community-based design template and describe the adoption by the community including a high-level review of current data assets, publicly released datasets, and web analytics. Although odc-sci.org is in its late beta stage of development, it represents a successful example of a specialized data commons that may serve as a model for other fields.

## Introduction

In the last decade, the need for more openness and transparency in scientific research has become apparent, with calls from funders, journals, lawmakers, researchers, and the general public to improve the self-correcting nature of the scientific literature. The fact that many experiments are not published, the lack of transparency of published studies, and the difficulty of accessing the source data underlying published results have been recognized as significant barriers to reproducibility (Chan et al., 2014). Data openness and sharing are potential solutions to deal with some of these barriers and increase the value of research by providing direct access to data for conducting replication and large-scale pooled studies at the subject level (Ferguson et al., 2014). Although the benefits of sharing research data have long been recognized within computational fields such as imaging neuroinformatics, ‘omics and clinical informatics (Kennedy, 2012; Piwowar et al., 2007; Pronk et al., 2015; Roundtable on Environmental Health Sciences et al., 2016), building the necessary social culture and infrastructure for data-sharing in other fields with non-standardized heterogeneous preclinical data of diverse types (‘long-tail’ data) remains a challenge (Borgman, 2012; Callahan et al., 2017; Ferguson et al., 2014; Roche et al., 2014; Tenopir et al., 2011). Relatively recent movements calling for accountability and transparency, such as open science, are challenging the traditional scientific dissemination establishment based on scientific narratives in papers. These movements have generated new forms of academic merit-based data sharing that contest the culture of data protectionism. At the same time, the lack of dedicated digital infrastructures has created a ‘semi-adoption’ of data sharing. For example, there has been an increase in the number of digital platforms for massive digital sharing, and some journals have started hosting data files. There is no doubt that these all-purpose solutions are better than non-sharing, but increasing evidence suggests that “data deposition” without curation and proper documentation might not be sufficient for achieving the goals of data sharing for reproducibility. In order to accommodate every possible need, all-purpose data repositories impose little or no requirements on file format, data structure, and documentation, making it difficult to reuse and integrate these data with other data resources. Additionally, such data repositories can create a false sense of accomplishment where researchers believe they have contributed in data sharing. However, if the data is not actually interpretable by humans, digitally interoperable by software systems, and reusable, the act is ultimately insufficient despite superficially appearing in-line with the cultural movement towards open data and open science. Studies based on game theory suggest that data sharing might be beneficial if a collaborative approach is taken and data sharing is embraced as a community rather than by individuals (Pronk et al., 2015). Thus, there is a need for solutions that elevate the quality and value of shared data for reusability which can be achieved through dedicated data services and collective efforts for specific research communities.

An important step forward is the cultural adoption of the ‘FAIR data principles’ (Wilkinson et al., 2016), a set of recommendations establishing a framework for data sharing stating that data should be Findable, Accessible, Interoperable and Reusable (FAIR). The first two are relatively easy to implement technically although they do require a cultural shift for the research community to embrace data sharing. The all-purpose data sharing platforms do a great job of making shared data findable and accessible, offering solutions for most researchers and lowering the bar for cultural adoption of data sharing. However, increasing the utility of shared data and FAIRness requires the hosted data, and data-related resources, to be interoperable and reusable. Achieving these latter two principles requires overcoming additional engineering and data management challenges atop cultural adoption. Community-driven and problem-specific infrastructures can overcome both the sociocultural and the technical challenges to achieve FAIR share data. However, community acceptance and financial support are essential.

Here, we report on the adoption of FAIR data principles by the field of spinal cord injury (SCI) research, offering an example of sociocultural and technical embracement of data sharing and FAIR data principles by a specific research community. Preclinical SCI research produces diverse neuromotor recovery behavioral measures in rats, mice, nonhuman primates, and pooled de-identified human data. These neuro-behavioral data are often combined with histopathological ratings of postmortem tissue, and variety of molecular endpoints with data often collected in an ad hoc fashion in the same individuals over time (e.g., Ferguson et al., 2013; Kyritsis et al., 2021). Both clinical and preclinical research have worked to promote the cultural adoption of data sharing and standardization in the SCI research community after many years of collective action. For example, the creation of clinical SCI data repositories such as the National Spinal Cord Injury Model System Database (www.nscisc.uab.edu) in 1973 (DeVivo et al., 2002), the European Multicenter Study about Spinal Cord Injury (EMSCI - emsci.org) in 2004 (Curt et al., 2004) have been instrumental for the community to understand the value of data gathering and integration. More recently, the International Spinal Cord Society (ISCoS), the American Spinal Injury Association (ASIA), and the National Institutes of Neurological Disorders and Stroke (NINDS) joined efforts to develop standards such as common data elements (CDEs) for the collection and reporting of clinical research data (Biering-Sørensen et al., 2015; Charlifue et al., 2016). The pre-clinical SCI research community similarly gained valuable collective experience leading to the current stage of data sharing. The NINDS funded projects Multicenter Animal Spinal Cord Injury Study (MASCIS) in the 90’s (Basso et al., 1995, 1996; Young, 2002) and Facilities of Research Excellence in Spinal Cord Injury (FORE-SCI) in the 2000’s (Aguilar & Steward, 2010; Anderson et al., 2009; Steward et al., 2012) led to the development of standards and procedures for SCI research in current use across the globe. The events preceding FAIR sharing in pre-clinical SCI research have accelerated in the last decade, resulting in the development of minimal reporting expectations for preclinical SCI research (MIASCI)(Lemmon et al., 2014), a knowledge base and ontology for integration of SCI research data compatible with terminology standards (RegenBase) (Callahan et al., 2016), and the curation of the Visualized Syndromic Information and Outcomes for Neurotrauma (VISION-SCI) multicenter, multi-species SCI dataset (Nielson et al., 2014). It is noteworthy that these efforts tackle diverse data types beyond those covered under standardized imaging modalities supported by the Brain Imaging Data Format (BIDS) (Gorgolewski et al., 2016), ‘omics data standards (Chervitz et al., 2011), clinical physiological data standards such as Neurodata Without Borders (NWB)(Rübel et al., 2019; Teeters et al., 2015), and health informatics such as Observational Medical Outcomes Partnership (OMOP) standards.

In parallel to these efforts, some important events have generated momentum for a cultural shift in biomedical research in general: the acknowledgment of a reproducibility crisis (Begley & Ioannidis, 2015; Ioannidis, 2005; Macleod et al., 2014; Schulz et al., 2016; Steward et al., 2012), a lack of translation of preclinical research into clinical care (Lammertse, 2013; Seyhan, 2019), and the growth of the open access and open science movements (Laakso et al., 2011). In the SCI community in particular, important events include: (1) the success of VISION-SCI in recovering and repurposing data for new discoveries (Nielson et al., 2015); (2) the development of FAIR data principles (Wilkinson et al., 2016); (3) the endorsement of FAIR by NIH and other funding agencies; (4) the Craig H. Neilsen Foundation awarding the project that seeded the ODC-SCI (“Open Data Commons for Spinal Cord Injury Research” in 2016 to ARF); (5) the generalized support of funders to the ODC-SCI effort (Wings for Life Foundation, International Spinal Research Trust, Rick Hansen Institute, the US Veterans Affairs, the Department of Defense Congressionally Directed Medical Research Program) (Fouad et al., 2020); and (6) the SCI 2020 meeting hosted by NINDS. These events have been key elements in bringing the SCI research community together, providing the cultural environment that has ultimately allowed for the development of FAIR data sharing in SCI research.

Based on this prior work, the community has directly embraced FAIR data sharing by developing and launching the Open Data Commons for SCI (ODC-SCI, odc-sci.org), a platform to share tabular data of research in the field of spinal cord injury. This included the development of a leadership plan with term limits, orderly leadership succession, and proactive change management (Callahan et al., 2017; Fouad et al., 2020). The ODC-SCI is a community-based data sharing infrastructure with the goal of democratizing SCI research data by allowing users to access existing data, contribute new data, and utilize and create user-friendly tools for analytics and SCI knowledge-discovery all within FAIR guidelines. The goal of the present paper is to provide historical context and illustrate how members of research communities can work together toward the development of dedicated data sharing initiatives under the umbrella of FAIR. Our major conclusion is that development and adoption of FAIR principles by a research community may require several years of collective effort by multiple stakeholders.

## Methods

**Fig. 1:**
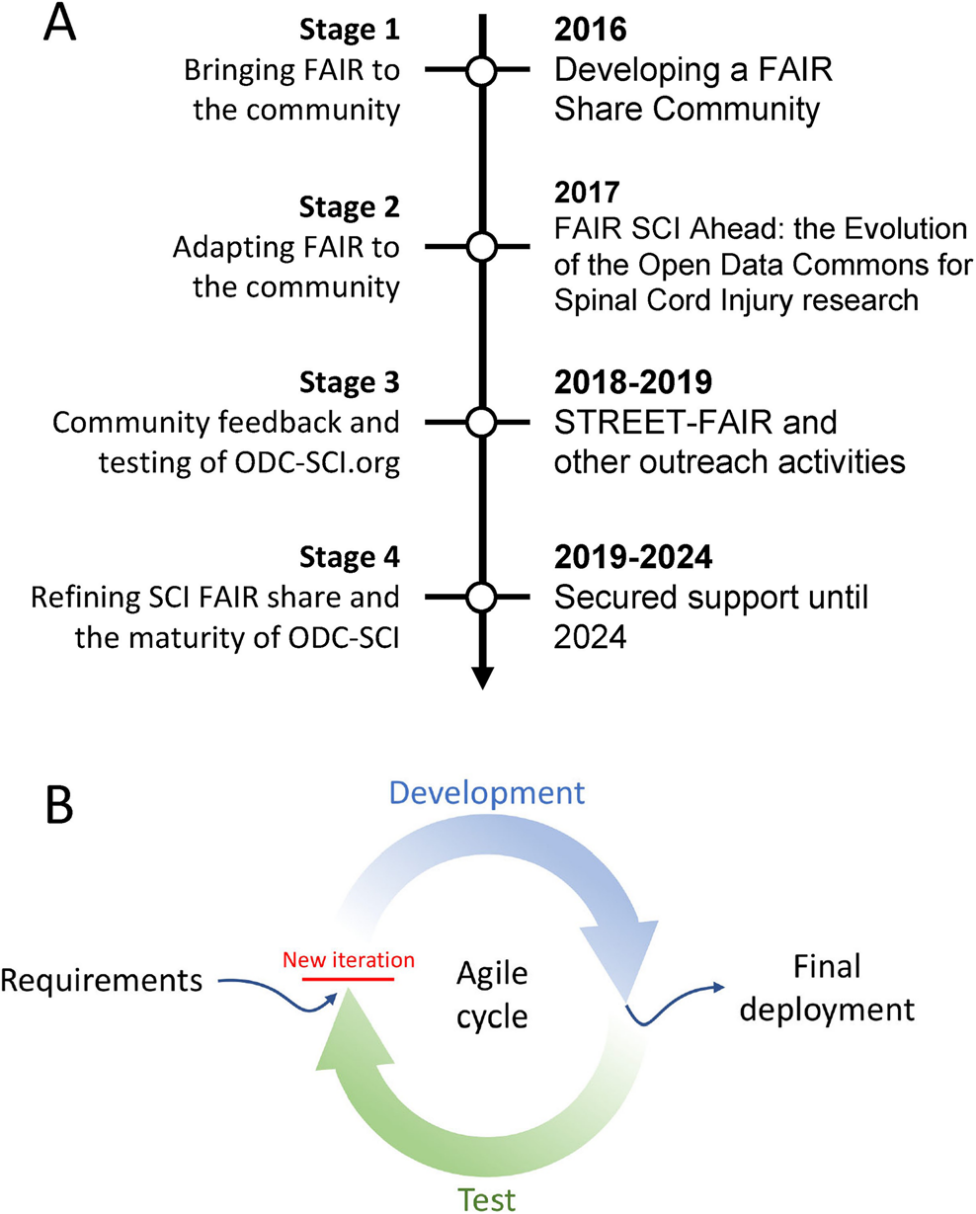
Staged development. We have divided the process by which the ODC-SCI and the SCI data-sharing community has come together in 4 stages (**A**). The three first stages seeded the foundations for ODC-SCI and stage 4, that has recently started, will bring ODC-SCI to maturity. During these stages the engagement with the SCI data-sharing community and the development of tools has occurred in parallel, in both cases using agile design principles (**B**). These consist on performing a requirement analysis (e.g., ask the community what data needs to be shared), followed by a period of design and development of tools and policies, and a period of feedback (testing) by the users and the community. When the implementation satisfies the requirements, the new functionalities can be incorporated to the ODC-SCI

The process of bringing the SCI community together around FAIR is an ongoing continuum, but we have conceptualized the set of events in three stages of community involvement using agile design principles (Fig. 1): (1) bringing FAIR to the SCI community; (2) adapting FAIR to the specific challenges of SCI research; and (3) responding to community feedback. Moreover, a fourth stage of establishment, consolidation, and maturity has recently been ensured by new funding (“Facilitating SCI research, translation and transparency: Going Public with the Open Data Commons”) through a multi-agency funding mechanism (KF, contact PI) and the continuous support of multiple stakeholders (SCI foundations, SCI community organizers and advocates, publishers, governmental agencies, industry representatives, among others) (Fouad et al., 2020). Below we detail the stages and how they came about.

### Stage One: Bringing FAIR to the Community

In 2016, the NINDS in collaboration with the ODC-SCI consortium hosted the “Developing a FAIR Share Community” workshop with different representatives of the SCI community to discuss data sharing in SCI (Callahan et al., 2017). The workshop was co-sponsored by the NINDS and the University of Alberta, with contributions from the International Spinal Research Trust, the Rick Hansen Institute, Wings for Life, and the Craig H. Neilsen Foundation. The goal was to have an open conversation with different SCI stakeholders about the readiness of the field for the challenge of data sharing and to develop a path toward adopting FAIR in the SCI community. The development and outcomes of that meeting are thoroughly discussed elsewhere (Callahan et al., 2017), though a summary of the conclusions are of interest here. The SCI community was receptive to data sharing at the time the workshop took place. This was demonstrated by polling responses that suggested the willingness of the participants to share data to some degree and by the collective efforts preceding the meeting as described above. However, there was disagreement on how open or restrictive sharing should be (i.e., available to the public or under access control). Major challenges and needs towards adopting FAIR were also identified. Community members voiced concerns about: (1) the added time required for sharing data, (2) the lack of specific funding mechanisms for researchers to practice data sharing, (3) the absence of dedicated infrastructures for sharing, discovery and reuse of SCI data, (4) the need for better standards allowing for augmented interoperability across the community, (5) the implementation of mechanisms to protect the intellectual property of the data owner, (6) the proper attribution allowing for data citation, and (7) the need to ensure the stability of the system.

The result of the 2016 meeting established a roadmap for FAIR adoption by the community. The development of the odc-sci.org platform moved forward to respond to the needs expressed by the community and establish the initial requirements to operationalize FAIR. At the same time, a steering committee was formed with individuals representing different sectors of the community to oversee the development of ODC-SCI. Moreover, broader community thoughts and ideas continued to be collected through outreach activities and presentations in scientific meetings about ODC-SCI and FAIR data sharing in SCI after the 2016 meeting.

### Stage Two: Adapting FAIR to the Community

One year after the first meeting, the “FAIR SCI Ahead: the Evolution of the Open Data Commons for Spinal Cord Injury research” workshop took place as a satellite event during the 2017 SFN (Society for Neuroscience) meeting in Washington DC. This second meeting was co-sponsored by Wings for Life, International Spinal Research Trust, Craig H. Neilsen Foundation, and NINDS. The goal was to discuss fundamental questions with the SCI community about how to adopt FAIR data sharing and develop specific policies that govern sharing in the community. These questions were derived from the challenges the community expressed during the first meeting, with the intention to create a concrete list of actions toward FAIR data sharing. The detailed results of this second meeting are documented in Fouad et al. 2020. Briefly, the community-driven recommendations can be summarized as: (1) the data to be shared should be individual-subject data in tabular form that underlie analysis rather than the raw data (e.g., images in histological analysis), in order to balance the technical and sociocultural challenges. Such data could also include data from ‘failed’ experiments that are difficult to publish because of publication bias (Macleod et al., 2009; Watzlawick et al., 2014, 2019). (2) The user permissions and rights that describe who can view and use the data needs a flexible policy, allowing for different members of the community to adapt the system to their specific needs. (3) Data curation and quality control processes should be put in place with enough flexibility to accommodate different goals that members of the community might have when sharing/accessing data. The creation of a ‘curation board’ formed by members of the community with expertise in SCI research was suggested. (4) The community felt that there is a need for ‘minimum information’ metadata allowing users to understand the shared data at a high level. Similarly, the adoption and augmentation of existing standards like MIASCI (Lemmon et al., 2014) and the RegenBase ontology (Callahan et al., 2016) would be useful. (5) Users should gain credit for data sharing efforts while ensuring that this does not hamper the utility of the data. A license that legally binds the data (re)-user to give appropriate credit to the data creator (e.g., creative commons CC-BY) was recommended by the community. (6) The use of digital object identifiers (DOIs) were approved as a viable mechanism to generate citable units that would credit researchers for sharing their data.

### Stage Three: Community feedback and Testing of odc-sci.org

With the general agreements regarding issues such as models of data access, quality control, and licenses in place, the ODC-SCI team implemented features into the platform to accordingly realize the vision that the SCI community understood as FAIR and found acceptable for data sharing. After several months of internal testing, a beta release (a version to be tested by users outside of the developing team) was made available during 2018. During the first period of odc-sci.org testing with a small group of external users, it rapidly became apparent that guiding users through the structure, functionalities, and workflow of the odc-sci.org was not an easy task. There was a notable learning curve associated with understanding the process and navigating through the site (e.g., from registering an account to uploading data and applying for a DOI). Members of the development team had to dedicate time to explain the ODC-SCI portal to users individually, which quickly created a bottleneck for utilization. In order to reach a broader audience and encourage more members of the SCI community to join the FAIR share movement, a third workshop entitled ‘SCI Team Research Enabling Expansion and Translation of FAIR’ (STREET-FAIR) was held as a satellite event at the 2018 SFN Neuroscience meeting. The STREET-FAIR was supported by the International Neuroinformatics Coordinating Facility (INCF) with the goal of promoting FAIR data sharing principles to the SCI community by (1) providing an update on the ODC-SCI portal and its support for FAIR data sharing and (2) encouraging participation in the odc-sci.org while offering one-on-one guidance for the participants to explore the portal and progress on their way to sharing data. To make the session practical and interactive, participants were challenged to use their own data as a working example in a hackathon-style format.

Upon initiation of the workshop, we realized that the ODC-SCI system was not prepared to handle the volume of network traffic of all the participants at once. The problem was rapidly detected and corrected on-site but clearly highlighted the value of the massive demonstration/work for beta testing the site to reveal unforeseen problems. Other challenges that were found during the course included technical bugs. For example, one participant observed that the ‘0’s in a dataset were transformed to blank cells in the uploaded data as a result of the ODC’s upload process misinterpreting values of “0” as missing data. Others mentioned the difficulty when using specific web browsers, pointing towards software compatibility issues. These and other technical issues raised during the workshop were rapidly corrected in updates to the platform following the conclusion of the meeting. Moreover, participants pointed out the need for improving self-explanatory tutorials and help materials that would facilitate the learning experience for those who could not attend the workshop. Notably, beyond identifying points for improvement, the workshop provided opportunities to stress test more uncommon features. For instance, participates who did not bring their laptops instead accessed the ODC-SCI through smartphones and tablets and helped verify that the site was functional on mobile devices and browsers.

The organizers and participants of the meeting concluded that having educational hands-on workshops is an instrumental tool for bringing awareness of the FAIR data sharing efforts to the broader community. Equally important, getting direct and indirect feedback (i.e., gathering opinions or watching users interact with the system using their own data) from community members representing different users with different goals is essential for the success of the collective effort. It is important to stress that working on their own data rather than test, users are likely to be more engaged and may notice errors in the systems more readily. The impact of conducting the STREET-FAIR meeting is evident in both the increasing usability and robustness of the system, as well as in community adoption.

During the first quarter of 2020, a second spate of updates for the ODC-SCI platform took place to implement the basic functionalities in response to the needs of the community. Moving forward, we implemented an user-centered design approach to improve the user experience and usability of the workflows and the site. The ODC-SCI team engaged in focus meetings with fast iterations between workflow implementations and user feedback that generated a new design guided by the users. An updated ODC-SCI site was made available in April 2020 based on these sessions with future updates planned as we are moving to the fourth stage of bringing together the SCI community around FAIR sharing.

### Stage Four: Refining SCI FAIR Share and the Maturity of ODC-SCI

The culmination of these three stages reflects the completion in 2019 of the Craig H. Neilsen Foundation funding that seeded the development of the ODC-SCI. However, much work remains to be done. The current version of the odc-sci.org has implemented the basic functionalities that translate most of the needs and policies decided by the community. Nonetheless, advanced functionalities such as incorporating tools for increasing interoperability and reusability and more challenging policies and procedures such as curation or establishing a sustainability model are still works in progress. A new multi-agency award supported by Wings for Life and Craig H. Neilsen Foundation entitled “Facilitating SCI research, translation and transparency: Going Public with the Open Data Commons” ensured funding for the next 5 years. Moreover, we have in-kind support from International Spinal Research Trust and other funders supporting data sharing moving forward. The main goals for this new phase are to advance the development of the odc-sci.org, to continue outreach, and to consolidate the FAIR community effort that will help release the full potential of data sharing in SCI research. Specifically, the project plan foresees the implementation of quality control and curation processes, the development of tools for better data searching and discovery to improve data findability and reusability, and the mechanisms for continuing community outreach and education.

During the initial phase of this new grant, we formalized the governing structure of the ODC-SCI and divided the organizational structure into different teams or boards: a Leadership team to coordinate the development and operation of ODC-SCI, an Executive board to offer oversight and be involved in executive decisions, a Community board to engage with the community and to receive community feedback through workshops, and a Data Science team to be responsible for data curation, quality control, and revision. The constituents of the Executive and Community board and some of the Data Science team are members representing the broad and heterogeneous SCI stakeholder community with the commitment to serve a 3-year term. Setting term limits gives the opportunity to rotate between constituents, allowing for new ideas and visions from a rapidly changing community.

In addition to maturing the ODC-SCI community portal in this stage, we are formalizing the implementation of the odc-sci.org using user-centered design practices. This has created a first major update of the odc-sci.org website with a stream of updates that will be released as new processes and features are incorporated. The following section offers an overview of the current implementation of odc-sci.org.

## Results

The odc-sci.org system has been designed and operationalized as a framework that aligns the necessities of the SCI data-sharing community to FAIR share principles through inter-connected functionalities. The process by which the community was engaged is described in the "Methods" section.

### ODC-SCI Data Spaces and Account Types

**Fig. 2:**
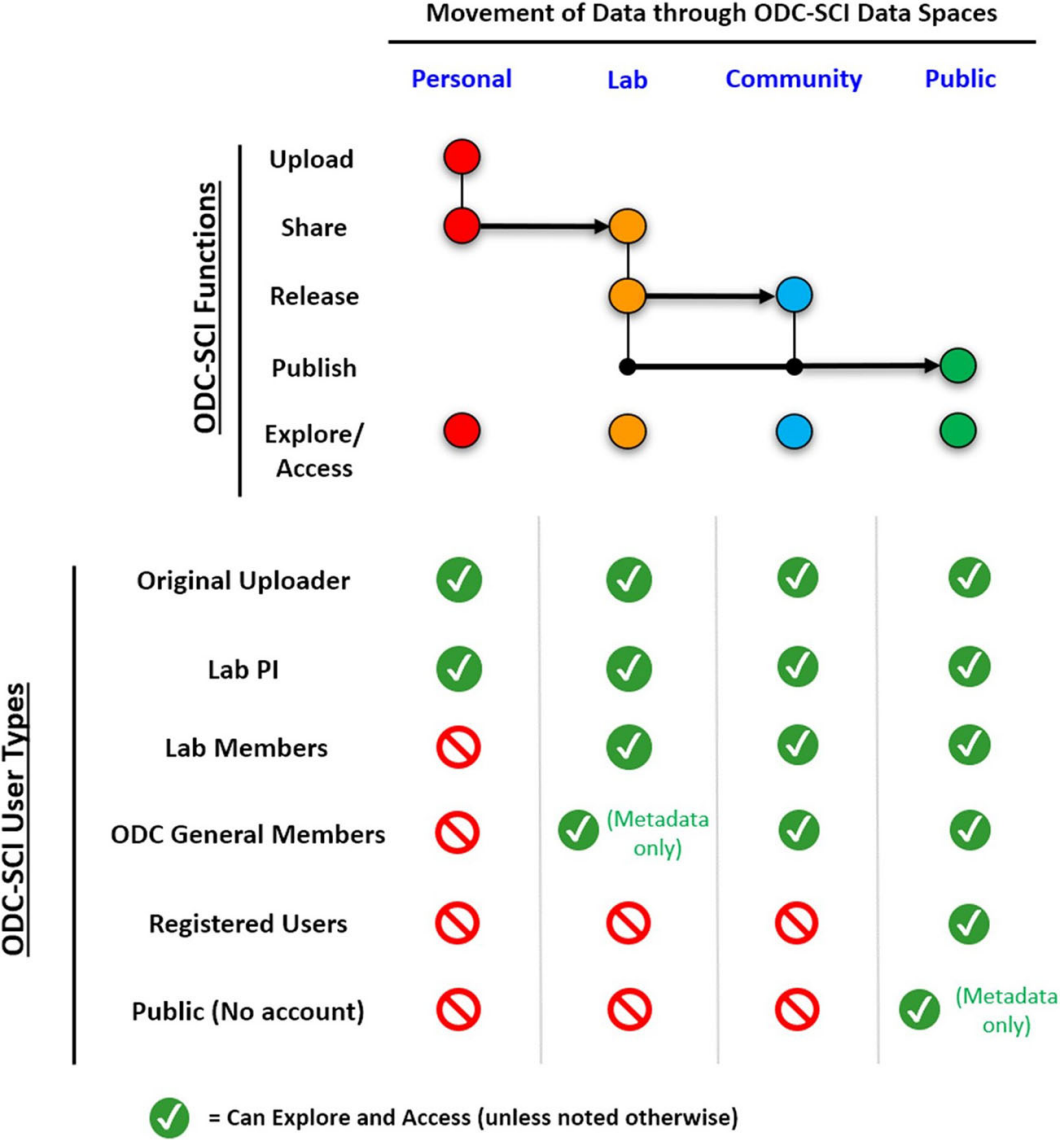
ODC-SCI data spaces and movement of data. Data on the ODC-SCI can live in different data spaces on increasing order of privacy. The Personal space is the most private space where users (Registered users who are part of a Lab) can upload data, share their uploaded data with their Lab (after PI/Lab manager approval) and explore and access data that is present in the user Personal space. Datasets at the Lab space can be explored and accessed by all users who are members of the same Lab. In addition, PIs and Lab managers can release the data to the ODC-SCI Community space or request DOI for publication. Datasets that are released to the ODC-SCI Community space can be explored and accessed by any registered user who has a Community member account (eighter general members or members of a laboratory). From the Community space, datasets can also be published by requesting DOI. This tiered system is hierarchical, since a dataset that for instance is released to the Community space, is still present in and belongs to the original Lab space and uploaders

The SCI data-sharing community has designed an incremental process for releasing and publishing data where data is first shared on a limited basis and then made progressively more open before final release (i.e., publication). The platform is accordingly built with hierarchical spaces for datasets. Each space determines who can access the data (Fig. 2). When a dataset is initially uploaded into the personal space, it is only accessible to the uploader and the PI of the uploader’s lab (user roles are explained in the next section). The successive levels of sharing will finally reach a public space; where at the discretion of the PI, users can publish their dataset with a creative commons (CC) license and a digital object identifier (DOI, Fig. 2). Published data is then accessible to any registered user of the ODC-SCI and does not require the audience to be part of the ODC-SCI community.

**Fig. 3:**
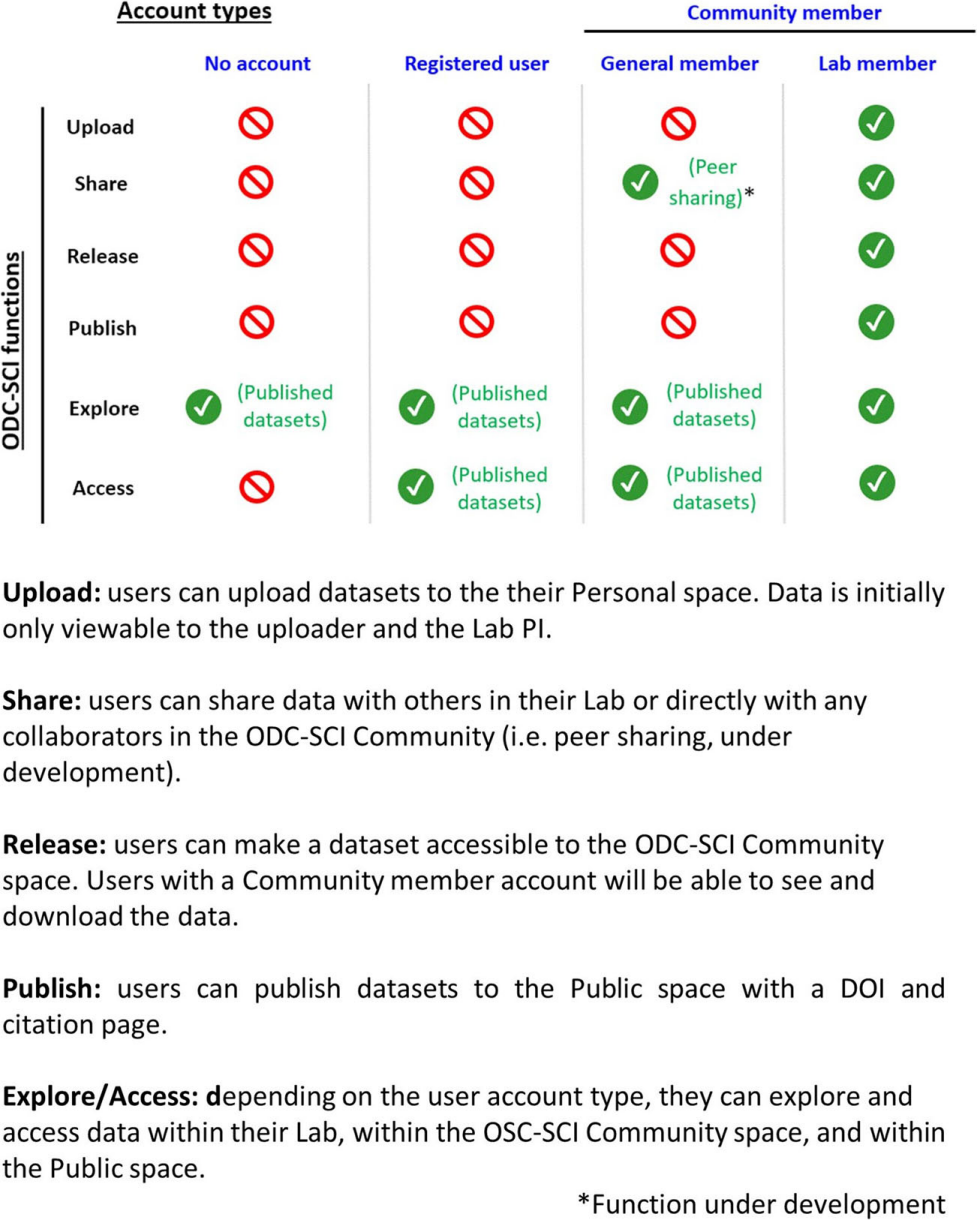
ODC-SCI account types and functions. Access to different functions on the site are determined by the account types. Visitors to the platform with no account can only explore the metadata for published datasets but can not see nor download the data. Registered users who are not part of the ODC-SCI Community can explore and download published datasets. Registered users who become part of the ODC-SCI Community will be able to explore and download published datasets, as well as get private peer sharing (feature still under development). To gain access to all the full suite of functions users will have to be part of a Lab in the ODC-SCI

The platform is designed to reflect community concerns and agreements reached in the above-described workshops about when data would be shared and with whom. Four different account types define the actions a user can perform (see Fig. 3) built upon the Neuroscience Information Framework (NIF)/SciCrunch technology stack developed by the FAIR Data Informatics Laboratory at University of California, San Diego. Becoming a registered user requires signing in by providing a valid email (preferably an institutional one) and agreeing to the terms of use of the site. Registered users can request to become ODC-SCI Community members with further approval by the Leadership team. Two types of Community members are defined. The most permissive account type is becoming an ODC-SCI Community member associated to an ODC-SCI lab, known as a Lab member. Community members can request to be part of an ODC-SCI lab and/or create their own Lab. The user obtains a Lab member account type upon approval of this request by the respective ODC-SCI lab PI or Leadership team. Lab members can perform all actions in ODC-SCI. Users approved for a Community member account but not associated with a specific ODC-SCI Lab can explore and access public datasets and share data peer-to-peer (feature in development). For Lab members, three possible permission levels are defined for each ODC-SCI Lab to which the user has access: regular lab member, lab manager, and principal investigator (PI). Regular lab members can only act on their own datasets or share their dataset to the private laboratory space. Lab managers have the same privileges as regular lab members but can also manage the laboratory space (i.e., accept new members, approve data to be shared to the laboratory and community spaces, or request a DOI for publication). The highest level of authority is the PI; PI’s have full control of their laboratory space including managing lab users and sharing datasets beyond the laboratory with the entire ODC-SCI Community or publishing their data to the Public space. PI’s also have the additional privilege of being able to assign the PI status to others or change the permissions of any members of their lab. In any given virtual laboratory, several members, managers, and PIs might coexist, even if they are from different research groups. This approach allows for multi-lab or multi-center data sharing in a private setting by creating a virtual lab on the ODC-SCI to manage the collaboration.

### ODC-SCI Data Format and Upload

**Fig. 4:**
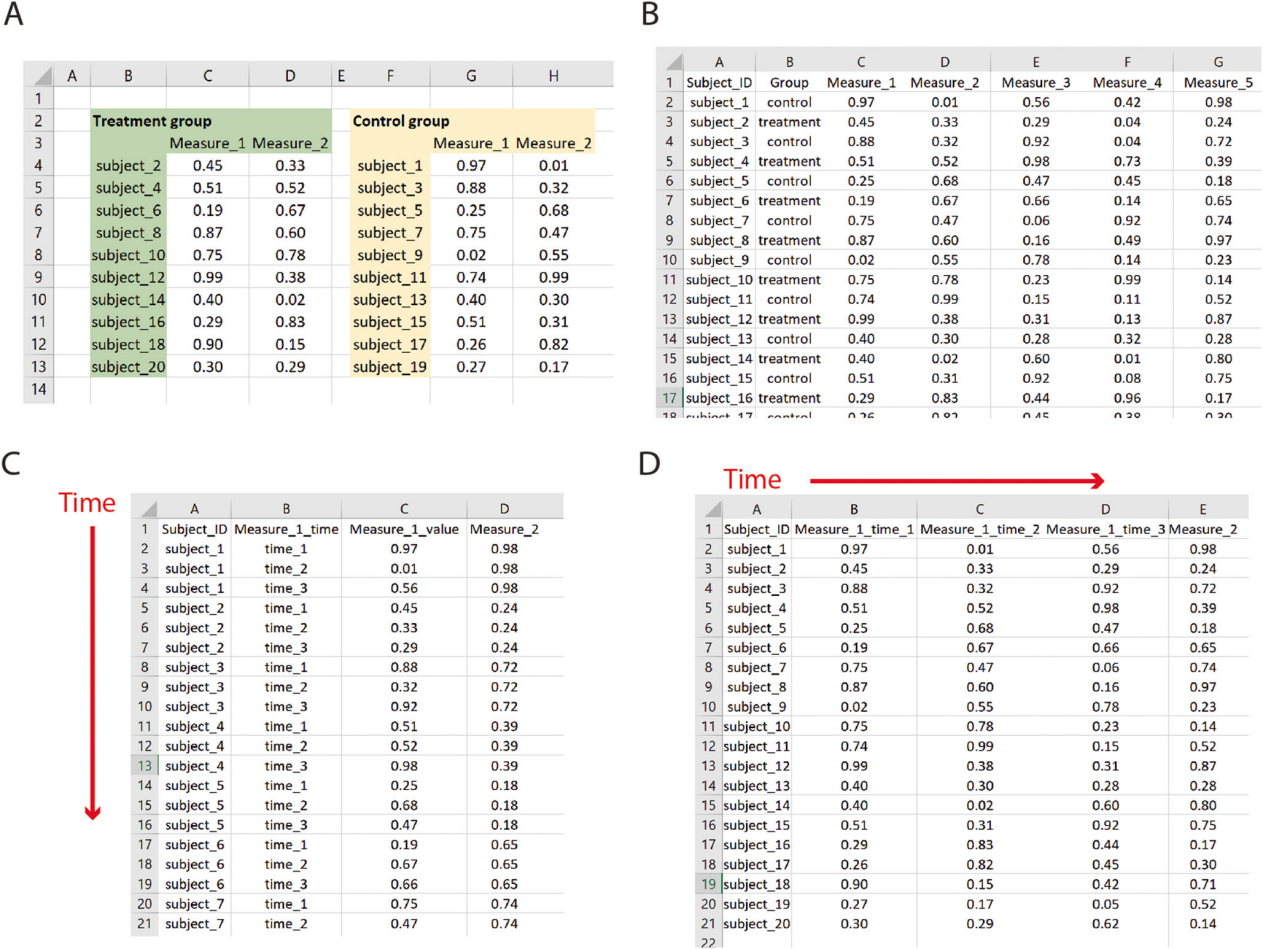
Machine vs. human readable tabular formats. How data is formatted into spreadsheets can affect the readability of it. As humans, we benefit from visual clues such as blank spaces or colors and from complex data organizations that divide data into chunks (e.g., groups of subjects) (**A**). Although this formatting of the data can be self-explanatory for humans, the complexity and lack of a consistent structure across researches make it challenging to generate standards that can be used by machines to process and understand data. The readability of a spreadsheet by a machine can be dramatically improved with simple rules (Broman & Woo, 2018) to organize the data in a structured manner (**B** to **D**). In ODC-SCI, data can be uploaded using spreadsheet type file (as .csv file) where columns are variables (also known as fields), the first row contain the variable names or headers and each consequent row is a unique record, meaning that there are not two identical rows on the dataset, and completely empty rows and columns are not allowed. The ODC-SCI database is organized around the subject identification number and thus it must always be present in the dataset. This formatting can have different variations depending on the hierarchical relationships between variables (such as in the case of repeated measures like time). For example, the same variables are collected at different timepoints, a time column can be specified, and subjects can be repeated in rows with records for each time point in different rows, known as semi-long format (**C**). Contrary, a new column can be created for every variable and every time point known as wide format (**D**), in which case each subject is only present in one row. When possible, ODC-SCI recommends using semi-long formats

The ODC-SCI incorporates various features contributing to the interoperability and reusability of data. First, the ODC-SCI standardizes the use of the comma separated value (*.csv) format for the data file upload process. We chose .csv because it is a widely used and open format that can be opened and edited in almost any data and text editor including spreadsheet/analytics software. This offers flexibility in terms of data organization and compatibility. These features allow for a balance between human and machine readability of the data, making it accessible for the research community while maintaining a level of machine interoperability and reusability (Fig. 4). Requirements for data formatting using the .csv files are easy to comply with: rows are observations, columns are variables, and the first row contains the headers or names of the columns. The ODC-SCI database is structured at the subject-data level, and therefore a unique identifier for each subject represented in the .csv file is required. Beyond that, current versions of the site do not impose further constraints on how to organize the data, giving flexibility for adapting to the user’s needs. During the process of uploading the .csv file to the site, a few automatic checks take place to ensure the minimal format requirements that would allow for integration of the data on the ODC-SCI database (Box 1). If a dataset does not pass this check, a notification is displayed pointing to the source of the issue. Once the user corrects the problem(s), the data can be uploaded to the site.

**Box 1:** ODC-S 534 CI data formatting quality checks.

Source errors (Checked at upload): ODC-SCI can read-in the data file.	
Structure errors: These errors are caused by formatting issues on the dataset	
- Blank-header (Checked at upload): There is a blank variable name. All cells in the header row (first row) must have a value.	
- Duplicate-header (Checked at upload): There are multiple columns with the same name. All column names must be unique.	
- Blank-row (Checked at upload): Rows must have at least one non-blank cell.	
- Duplicate-row (Checked for publication): Rows can not be duplicated.	
Schema errors: These errors reflect conflicts between the data dictionary and the dataset.	
- Extra-header (Checked for publication): The dataset contains at least one variable name not defined in the data dictionary.	
- Missing-header (Checked for publication): The dataset is missing at least one variable name defined in the data dictionary.	
- Missing-definition (Checked for publication): The definition of a variable in the data dictionary is missing.	
- Required-constraint (Checked at upload): A required field for the dataset contains no values or is not assigned on the dataset. Currently the only required value in the datasets is the subject identifier. As ODC-SCI develops additional data standards, it is possible that more variables will be required on all datasets.	
- Value-constraint (under development): The values of a variable should be equal to one of the permitted values enumerated in the data dictionary, or within the limits of the permitted values.

When a dataset is first uploaded, the site assigns a persistent identifier to the dataset, and the user is required to provide a title and a narrative description of the content. This information is displayed in the lab space together with the name of the user who uploaded the data which facilitates other Lab Members in finding the data. If the dataset is released to the ODC-SCI Community space, these metadata elements are displayed in their respective landing pages with the addition of the number of observations and records contained in the dataset and the name of the lab where the data was uploaded. This allows members of the ODC-SCI Community to find and identify datasets of interest. Members of the SCI data-sharing community have expressed the need for these minimal metadata information about a dataset to be present in the ODC-SCI Community space, even when data remains private in the Lab space. The ODC-SCI design team is contemplating this option for future implementation which would help inform users on what other datasets might become available or allow users to ask for private sharing while the data is limited to internal usage. Once a dataset is made public (see section below), a citation page is generated and a DOI issued.

### Publishing Datasets at ODC-SCI

Publishing datasets through ODC-SCI means making data available to the general public (with a regular registered user account) under an open source license (CC-BY 4.0) and with an associated DOI which generates a citable unit. One of the goals of ODC-SCI is to promote FAIR data principles and reproducibility of the ODC-SCI data which requires a minimal standard for the datasets before it can be made public. To achieve such a standard, we have created a quality check and review process that is conducted by members of the ODC-SCI Data Science team. The refinement of this process is ongoing, although the basic workflow and tools are in place. As a requirement for publication, a data dictionary (i.e., ‘codebook’) must be provided. Specifically, the data dictionary gives definitions, units, permitted values, and other valuable information necessary for understanding the collected data. This type of documentation is essential for the reusability of the data but is often overlooked in general purpose repositories. Another important piece of documentation is the metadata information associated with the dataset. This is constituted by an appropriate title, a structured abstract with a description of the study purpose, an overview of the data collected and major conclusions of the study, a list of authors and contributors, a list of identifiers and links to other resources such as an associated paper, and funding information. An editable webform for each uploaded dataset can be used to provide this information directly on the odc-sci.org site.

The dataset, data dictionary, and metadata undergo structured quality checks to ensure minimal ODC-SCI standards before publication. First, datasets and data dictionaries are reviewed for potential inconsistencies in formatting and structure (Box 1) such as whether a variable is present in the dataset but is not defined in the data dictionary. Some of these checks are performed automatically during the dataset upload, and others are done by members of the Data Curation team while reviewing the dataset for publication. As the ODC-SCI progresses, we will develop automated tools for conducting all dataset and data dictionary formatting quality checks. The second part of the review process is an editorial revision of metadata information to ensure that it contains enough information to adhere to FAIR standards. Once the dataset, data dictionary, and metadata are approved by the Data team for release, a DOI and citation page will be generated and made public. It is important to keep in mind that based on community feedback this process has been put in place to ensure minimal quality of shared data for interoperability and reusability, and the review process increases the time to generate a DOI and make data public compared to general purpose repositories that may not provide curation services.

Once published, ODC-SCI adds searchable tags to a dataset by marking up the pages with structured vocabulary such as the one provided by schema.org. This permits the user to search for the dataset DOI, for the citation information, or for a related article in a search engine resulting in web links to the publicly shared dataset. Moreover, the ODC-SCI is part of the SciCrunch ecosystem (Whetzel et al. 2015) and is indexed as a resource (RRID: SCR_016673) that can be found by the Neuroscience Information Framework (NIF, neuinfo.org).

### Community Adoption and Web Analytics on the odc-sci.org

**Fig. 5:**
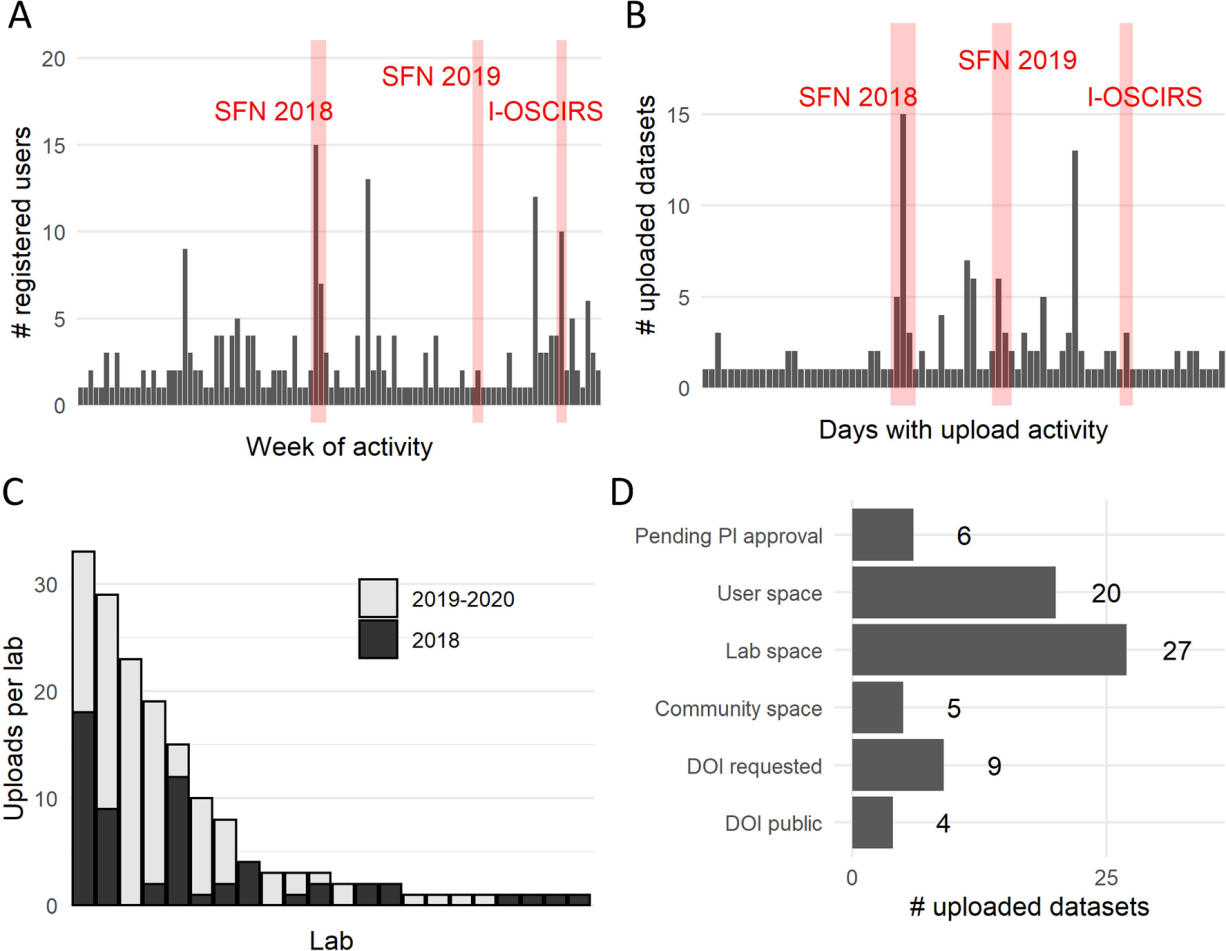
ODC-SCI activity. We tracked the activity on when users registered to the site (**A**), when datasets got uploaded (**B**), the number of uploaded datasets per Lab (**C**), and the status or data space where datasets are set (**D**). A total of 234 datasets have been uploaded. An estimated 38 % of the uploaded datasets are placeholder datasets created to explore the functionality of the portal, including datasets uploaded during development, test sets during outreach activities, and datasets by users who include “test” or “practice” on the description. Most of those datasets have been subsequently deleted and only active datasets are shown in **D**. Notice that although we have 11 requests for DOI at the time of writing, there are 2 datasets in preparation for being uploaded, and therefore not reflected in **D**

In order to evaluate the community adoption of the odc-sci.org, we derived aggregated metrics from the registered users (Fig. 5). From this information, we compiled descriptive statistics of new registrations, data uploads, and the spaces/stages where datasets are in the data release cycle (e.g., private space, lab space, DOI requested) to summarize the current data landscape of the ODC-SCI (Table 1). These metrics serve as a surrogate to study the adoption of the odc-sci.org and as an indicator of interest in data sharing by the community.

**Table 1:** ODC-SCI community activity as of July 2020

Registered users	248
Active datasets	103 with accumulated 1,379,988 rows and 18,359
Estimated individual subjects	N > 10,000
Total visits	4799
New visits	2649 from 46 countries
Recurrent visits	2150
Returning visitors	502* from 20 countries

*Note that the activity of the members of the development team are included in the returning visitors’ data, although the ODC-SCI team constitutes very few of those 502 visitors

As of July 2020, 248 users are registered on the site and 57 different laboratories across Canada, Europe and USA have been created. Some peaks of activity coincide with outreach and community workshops (Fig. 5B), where the maximum number of new user registrations in a week happened during the SFN Neuroscience meeting in 2018 where the STREET-FAIR workshop took place. Other activities such as the streaming at the International Online Spinal Cord Injury Research Seminars (I-OSCIRS; https://www.youtube.com/watch?v=LZ9DhxUUkeE&t=14 s) were also accompanied by an increase in the number of registered users. The number of uploaded datasets also peaked during outreach and workshop activities, although the increase in users is not always accompanied with an increase in uploads. In terms of the number of uploaded datasets per laboratory (excluding a test lab by the developing team), we observed a general trend of an increasing number of datasets from the same laboratory from 2018 to 2019–2020 (Fig. 5C). In addition, more laboratories were created in 2019–2020 with a corresponding increase in the number of labs that are uploading data to the ODC-SCI. Of the current active datasets that are not in the test laboratory, most are either in the private user space or internally approved to be shared in the private lab space (Fig. 5D). A total of 6 datasets have been released to the community, and 4 DOIs have been generated with the datasets available for the public (Ferguson et al., 2018; Liu et al., 2019; Puko & McTigue, 2020; Schmidt et al., 2019). At the time of publishing, 11 DOIs have been requested and are being processed, reflecting the commitment of the community to sharing data to the public. Importantly, the sequences of stages (private, to the lab space, to community, to public) is not necessary, and we have found that some authors choose to go directly from the laboratory space into DOI release upon completion of data curation and quality checks. To date, this type of lab-to-public sharing has largely been in the context of authors being asked by journals to provide a dataset DOI to coincide with the release of peer-reviewed papers, suggesting that publishers are starting to enforce data sharing policies and users are seeing ODC-SCI.org as a viable option to meet the requirements.

### Global Visits to odc-sci.org

**Fig. 6:**
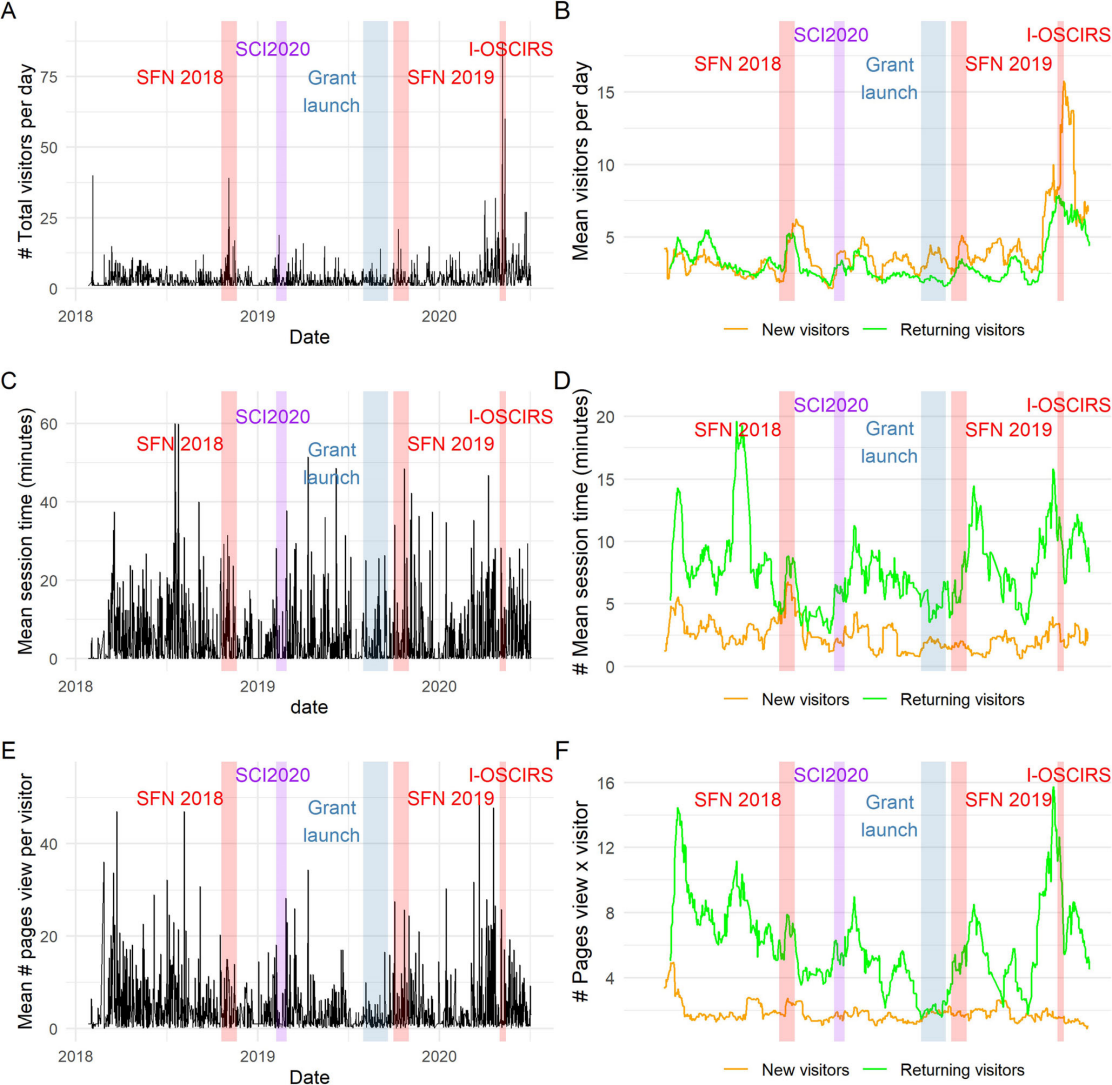
Traffic of visitors to the odc-sci.org. Using Google analytics traffic monitoring data we identified new and returning visitors over time (**A**-**B**), as well as the time spend per session in minutes (**C**-**D**) and the number of pages viewed per visitor/session (**E**-**F**). **A**, **C** and **F** show the raw daily metrics while **B**, **D** and **F** show 3 weeks moving average over the same period of time. Some of the important outreach activities are annotated on the graphs: SFN 2018 STREET-FAIR workshop, the SCI2020 meeting hosted by NINDS, the press release of the new multi-agency grant launch, the SFN 2019 ODC-SCI stand as part of NIF and the IOSCIRS online workshop

The odc-sci.org has been registered with Google Analytics since 2018, allowing us to measure usage and activity of the website. These tools do not allow for individual-visitor identification but rather aggregate metrics of usage of the ODC-SCI portal that can be used as indicators of community engagement to the site. One measure of global activity on the ODC-SCI is the number of returning and new visitors (Fig. 6A-B , Table 1). Fluctuations on the traffic of both new and recurrent visits can be appreciated where peaks of visitors can be associated to periods during or right after outreach activities. After most peaks of activity, traffic seems to return to an average baseline of around baseline level of around 2–3 new and 2–3 recurrent users a day on average. The average of new and returning visitors per day rapidly increased in the first and second quarter of 2020 with a particularly high traffic period during the I-OSCIRS seminar. It is too early to see if the latest jump in traffic will return to a similar baseline activity or if it will produce a new sustained base traffic with higher visitors on average per day.

**Fig. 7:**
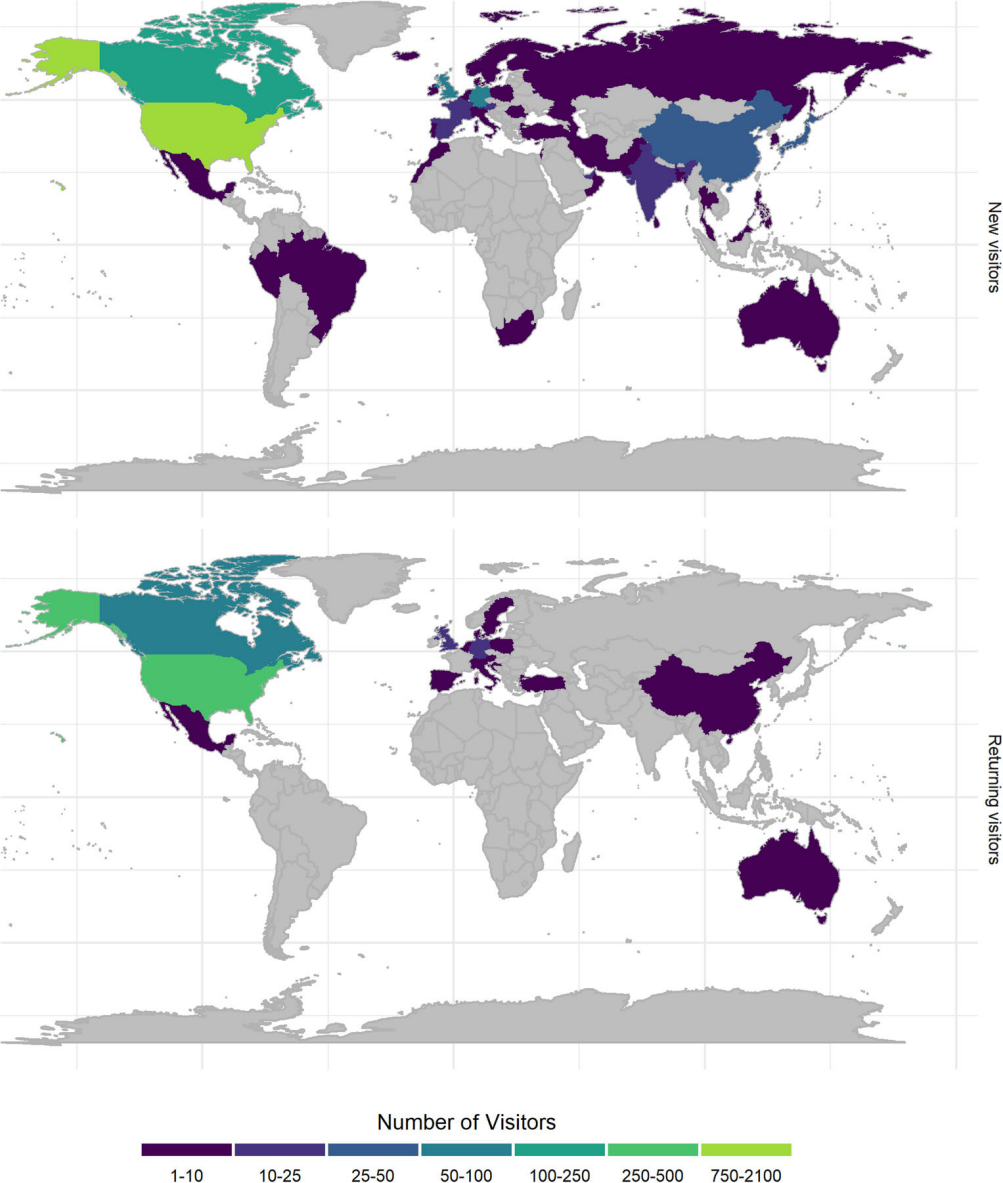
Geographical origin of internet traffic to the odc-sci.org. New and Returning visitors have viewed odc-sci.org since we started monitoring traffic

Two potential proxies for the level of engagement of visitors with the site is the time spent (Fig. 6C-D) and the number of pages viewed (Fig. 6E-F) per session. As a general trend, returning visitors spend more time and visit more pages than new visitors, which is to be expected since there is limited content that new visitors can access until they register as users. Thus, these two measures likely convey different things about whether the visitor is new or recurrent. New visitors who become registered users may come back after closing their session, and then be counted as returning visitor with more options on the platform. There are some peaks in the mean session time and number of pages viewed by visitors associated with hands-on outreach activities such as the STREET-FAIR workshop in 2018, but other activities did not register the same pattern as the I-OSCIRS seminar. This is similar to the fluctuations in new registered users and uploaded datasets, which suggest that different outreach activities produce different behaviors in visitors and users. The geographical locations of visitors indicate international traffic to the site (Fig. 7; Table 1).

## Discussion and Conclusions

The present paper highlights the journey to-date that the SCI research community has undertaken to adopt the FAIR principles to promote data sharing and research transparency in the context of heterogeneous preclinical research data types (‘long-tail’ data)(Ferguson et al., 2014). The data covered by the ODC-SCI enables FAIR sharing of data that falls outside of that covered by established standards such as clinical neuroimaging (e.g., BIDS), physiology (e.g., NWB), health informatics (e.g., OMOP). The experience of direct community engagement and application of agile design principles provides a template for achieving FAIR data sharing in a research community and may be repurposed in other research communities. We would summarize the steps taken as: (1) developing a history of collective and cooperative efforts around data collection and standards; (2) early assessment of the readiness of the community, the challenges, and the specific community needs while involving different parties to provide different perspectives on the data life cycle; (3) adapting FAIR principles to the specific needs of the community; (4) seeking community involvement and feedback, and combining it with agile design principles for constant iteration. Our experience to-date has led us to a set of fundamental principles for developing community-based FAIR technology (Box 2).

**Box 2:** Principles behind community-based FAIR data sharing technology.

- Emphasize community acceptance ahead of engineering perfection.	
- Recognize cultural change is slow and needs constant effort in parallel to offering technological solutions through a portal.	
- Multiple levels of research community engagement with multiple stakeholders (researchers, consumers, funders, government, publishers) are essential for evolving a data publication culture and the data platform.	
- Collaboration with funding agencies early on is essential and potentially the key for adoption of FAIR and open data sharing portals.	

To-date, this community-based, agile design framework has helped odc-sci.org meet the increasing requirements of journals and funders that published paper are accompanied by open and FAIR data that promote transparency and reproducibility of research. By embracing community engagement, our goal throughout has been to empower the community to meet such demands. The design philosophy has been to both adapt the design to user demands as well as guide them towards implementing FAIR principles by keeping a user-centric design that can accommodate different user types including primary data creators, reviewers and funders, and data consumers and meta-analysts. Overall, there has been an increase in the community usage of the odc-sci.org with important peaks of activity during major events, especially STREET-FAIR and I-OSCIRS. ODC-SCI has seen not only a constant increase of community members but also of registered laboratories and movement of datasets from the private space to the community and publicly accessible space. This suggests that the community of SCI researchers are beginning to use ODC-SCI and adopt FAIR data sharing principles. It is noteworthy that there this steady increase in traffic based on grass-roots interest without a large scale, coordinated marketing effort. However, a campaign to increase user engagement is planned as the portal moves into full production. In that regard, the metrics and numbers observed during the reported period will serve as a baseline to benchmark future development.

The next steps will involve further user-driven development of data dictionaries and standards that improve interoperability. Currently, data dictionaries are optional for datasets in private lab spaces but are required files (in .csv format) for all datasets released to the public. As the ODC-SCI is further populated and data dictionaries are uploaded together with datasets, we will be able to generate a list of variables or data elements that are commonly collected by the community. The dictionaries will thus ultimately inform the generation of the ODC-SCI common data elements (CDEs), a set of standards for variables that will help augment interoperability between data sources with the potential to include ‘translational interoperability’ of dataset across species. The effort would mirror the establishment of clinical CDEs for SCI by the NINDS (Biering-Sørensen et al., 2015). Notably, while there are preclinical standards being developed by NINDS workgroups for several disorders, there have yet to be directed efforts for preclinical SCI.

The ODC-SCI developing team is planning to incorporate functions to map the variables (i.e., data elements) present in the ODC-SCI data to existing data elements available through InterLex/NeuroLex (Larson & Martone, 2013), a dynamic lexicon of biomedical terms maintained by NIF. With the future creation of ODC-SCI CDEs and the integration of those through InterLex, the ODC-SCI will establish the tools for developing community standards and increasing interoperability in the SCI research field. We expect that these mapping functionalities, together with sufficient metadata and documentation, will provide a common data model with enough information for reusing the ODC-SCI datasets both by humans and machines. In time these may mature to the point that they can integrate with other, more mature clinical standards such as BIDS, NWB, and OMOP, among others.

As the SCI community has demonstrated, even in the absence of tightly defined knowledge engineering, it may be possible to extract new knowledge from semi-structured data if modern machine learning analytics are leveraged. Indeed, Nielson et al. 2015 and Almeida et al. (2021; in the present issue) demonstrates the utility of analyzing FAIR data even from archival laboratory data (25 years ago) to develop and externally validate new predictors of long term neuromotor recovery. The continuing development of the SCI data commons through odc-sci.org and broader neurocommons efforts will promote ever increasing knowledge through FAIR data sharing across individual researchers, laboratories, species, and perhaps even disease domains.

## Information Sharing Statement

The ODC-SCI is freely accessible under registration and approval as described in this manuscript.

## Data Availability

No applicable.

## Code Availability

No applicable.
